# A Rapid, Stability-Indicating RP-HPLC Method for the Simultaneous Determination of Formoterol Fumarate, Tiotropium Bromide, and Ciclesonide in a Pulmonary Drug Product

**DOI:** 10.3797/scipharm.1204-06

**Published:** 2012-05-22

**Authors:** Rakshit Kanubhai Trivedi, Dhairyshil S. Chendake, Mukesh C. Patel

**Affiliations:** 1 Analytical Research and Development, Integrated Product Development, Dr. Reddy’s Laboratories Ltd., Bachupally, Hyderabad-500 072, India.; 2 P. S. Science and H. D. Patel Arts College, S. V. Campus, Kadi-382 715, Gujarat, India.

**Keywords:** Method validation, Forced degradation, Method development, Assay, Aerosol drug product, Pressurized metered-dose inhaler, Chromatography

## Abstract

A stability-indicating reversed-phase high performance liquid chromatography (RP-HPLC) method was developed for the simultaneous determination of Formoterol fumarate (FOR), Tiotropium bromide (TRI), and Ciclesonide (CLS) in a pulmonary drug product. The desired chromatographic separation was achieved on the Zorbax SB C8, 5 μm (150 × 4.6 mm) column, using gradient elution at 230 nm detector wavelength. The optimized mobile phase consisted of a 0.2 % v/v perchloric acid as solvent-A and acetonitrile as solvent-B. The developed method separated FOR, TRI, and CLS in the presence of its five unknown degradation products within 10 minutes. The stability-indicating capability was established by forced degradation experiments and the separation of unknown degradation products. The developed RP-HPLC method was validated according to the International Conference on Harmonization (ICH) guidelines. This validated method was applied for the simultaneous estimation of FOR, TRI, and CLS in commercially available Triohale^®^ pMDI (Pressurized Metered-Dose Inhaler) samples. Furthermore, this method can be extended for individual estimation of FOR, TRI, and CLS in various commercially available pulmonary dosage forms.

## Introduction

The combination inhaler drug combines three essential medicines that are recommended for use in patients with severe Chronic Obstructive Pulmonary Disease (COPD). It contains a long-acting anticholinergic (Tiotropium), a long-acting beta-2-agonist (Formoterol), and an inhalable corticosteroid (Ciclesonide). Recent research shows that COPD is a multi-component disease and the individual components of combination pulmonary drug product target different aspects of the disease. Patients suffering from COPD who were prescribed combination inhaler drug product (Triohale^®^) will have to take just two puffs a day. This is a significant improvement over the current therapy involving two to three inhalers which can be confusing for some patients. Although there is no cure for COPD, the use of combination inhaler drug product will reduce symptoms, increase lung function, decrease the number of times patients are hospitalized, and lead to improved quality of life [1].

Several analytic methods have been reported for the qualitative and quantitative (either alone or in combination) determination of FOR, TRI, and CLS in pulmonary drug product formulations, by HPLC and other techniques [2–10]. A detailed literature survey for FOR, TRI, and CLS revealed that one analytical method is available using HPLC; Ravi Pratap *et al*. [11], describe simultaneous estimation of FOR, TRI, and CLS in pharmaceutical metered-dose inhalers. This reported method was not validated as per ICH guidance (for specificity, forced degradation study) and the limit of quantification is also high (4.32 μg/mL for FOR, 16.2 μg/mL for TRI, and 144 μg/mL for CLS). In the Triohale^®^ pressurized metered-dose inhaler, one spray label claim is 6 μg for FOR, 9 μg for TRI, and 200 μg for CLS. In Triohale^®^, TRI is 9 μg/spray and the published method claimed that the LOQ value for TRI compound is 16.2 μg/mL. Therefore, the published method [11] is not suitable for the quantification of the pulmonary drug product, Triohale^®^. In the same literature [11], limit of detection was 1.44 μg/mL for FOR and linearity was claimed to be from 0.72 to 8.64 μg/mL. Compound linearity is not possible below the limit of detection, which indicates that the published method work is not satisfactory. The same ambiguity was observed for TRI and CLS in the published method [11].

**Fig. 1:**
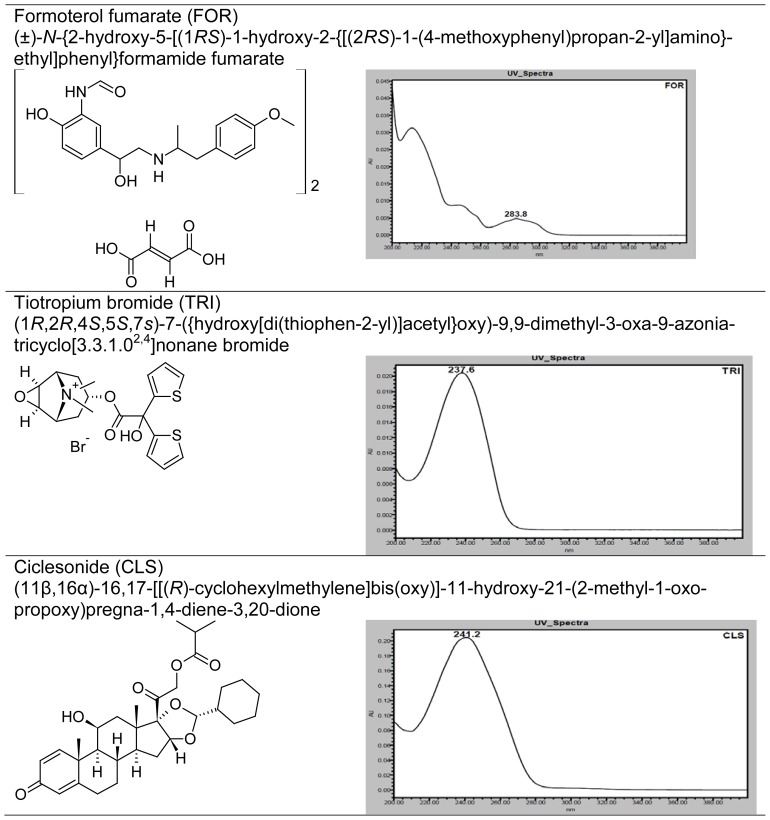
Chemical structures, UV spectrums, and chemical names of FOR, TRI, and CLS

The combination of FOR, TRI, and CLS is not official in any pharmacopoeia. So far, no RP-HPLC stability-indicating method has been reported for the rapid simultaneous determination of FOR, TRI, and CLS in pulmonary drug product. Therefore, it is necessary to develop a new rapid and stability-indicating method for simultaneous determination of three compounds (FOR, TRI, and CLS) in pulmonary drug product. The proposed method is able to separate FOR, TRI, and CLS from one another and from all five unknown degradation products within 10 minutes. Thereafter, this method was validated according to the ICH guidelines [12] and successfully applied for separation of all compounds of interest in the pressurized metered-dose inhaler (Triohale^®^, Cipla Ltd.). The chemical structures, UV spectrums, and chemical names of FOR, TRI, and CLS are presented in Figure 1.

## Results and Discussion

### Method development and optimization

The main objectives of the RP-HPLC method development for the rapid and simultaneous determination of FOR, TRI, and CLS in the pressurized metered-dose inhaler were: the method should be able to determine assay of the three compounds in a single run and should be accurate, reproducible, robust, stability-indicating, linear, free of interference from blank/placebo/degradation products, and straightforward enough for routine use in quality control laboratories.

The spiked solution of FOR (0.6 μg/mL), TRI (0.9 μg/mL), and CLS (20 μg/mL) was subjected to separation by RP-HPLC. Labeled claim of compounds and its working concentration is presented in Table 1. Various types of solvent-A and B were studied to optimize this method, which are summarized in Table 2 with their respective observations. Based on these experiments, FOR and TRI were selected as a critical pair for separation. Optimized HPLC parameters were; flow rate 1.2 mL/min; column oven temperature 25°C; gradient solvent program as per Table 3; 0.2% v/v perchloric acid as solvent-A and acetonitrile as solvent-B. In order to achieve symmetrical peaks for all substances and higher resolution between FOR and TRI, various stationary phases were also studied. Summary of stationary phases are presented in Table 4. Based on this summary, it was concluded that Zorbax SB C8 (150 × 4.6 mm; 5 μm) had a higher resolution (FOR and TRI) and more symmetrical peaks than all of the other compounds with respect to the other stationary phases and other equivalent columns. Column oven temperature was also studied, and found that 25°C was more appropriate with respect to separation. Based on compound UV spectrums, 230nm was found more appropriate for the simultaneous determination.

**Fig. 2:**
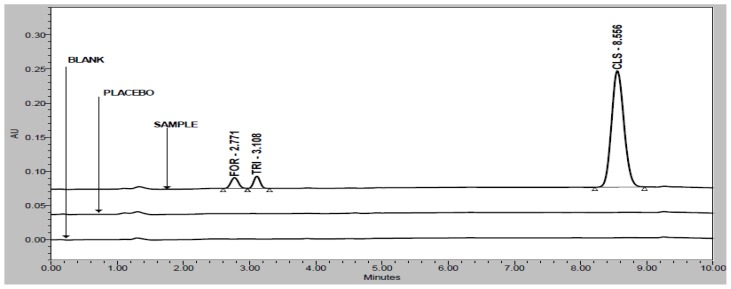
Overlay chromatograms of blank, placebo, and sample preparation

FOR, TRI, and CLS are well-resolved with each other and also well-resolved with all five unknown degradation products in the reasonable time of 10 minutes. There was not any chromatography interference due to blank (diluent) and placebo at the retention time of FOR, TRI, and CLS which is presented in Figure 2.

### Analytical parameters and validation

After satisfactory development of the method, it was subjected to method validation as per ICH guideline [12].

### Specificity

Specificity is the ability of the method to measure the analyte response in the presence of its potential impurities [12]. Forced degradation studies were performed to demonstrate selectivity and stability-indicating capability of the proposed RP-HPLC method. Figure 2 shows that there are no interferences at the RT (retention time) of FOR, TRI, and CLS due to the blank and placebo.

**Fig. 3:**
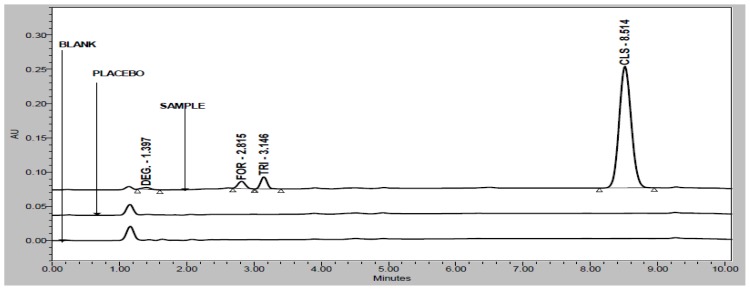
Overlay chromatograms of acid hydrolysis study

**Fig. 4:**
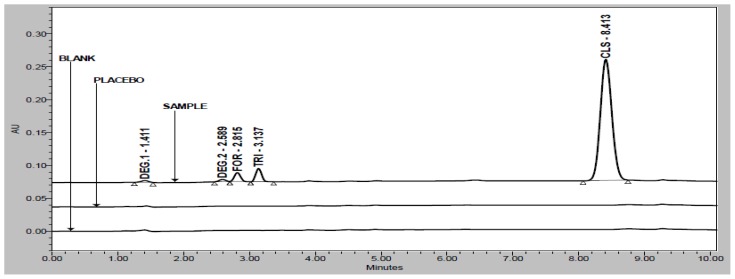
Overlay chromatograms of base hydrolysis study

**Fig. 5:**
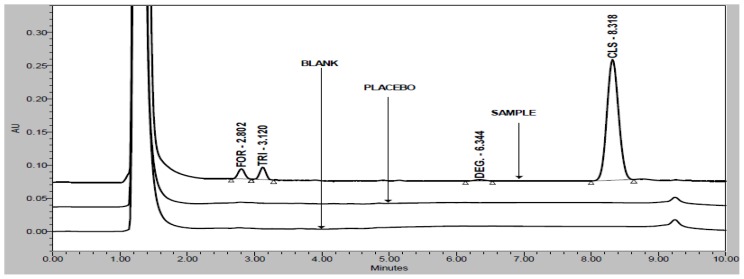
Overlay chromatograms of oxidation (H_2_O_2_) study

**Fig. 6:**
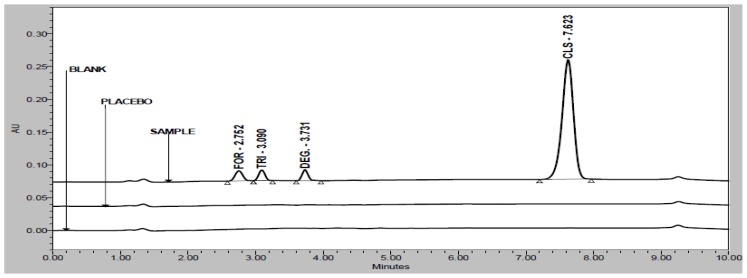
Overlay chromatograms of photolytic condition study

Degradation was observed when the drug product was subjected to acid hydrolysis (Figure 3), base hydrolysis (Figure 4), oxidative (Figure 5), and photolytic degradation (Figure 6). Peaks due to FOR, TRI, and CLS were investigated for spectral purity in the chromatogram of all the exposed samples and was found to be spectrally pure. Observations from the forced degradation study are given in Table 5.

### Precision

#### Instrument precision: (Suitability of system)

System suitability parameters were measured so as to verify the system performance. System precision was determined on six replicate injections of the standard preparation (Table 6). All important characteristics including % RSD (related standard deviation), resolution (between FOR and TRI), tailing factor, and theoretical plate number were measured. The percentage RSD of the area counts of six replicate injections was below 1.0 %, which indicates that the system is precise. The results obtained are shown in Table 6. All of the parameters complied with the acceptance criteria, and the system suitability was established.

#### Method precision: (Repeatability)

The precision of the assay method was evaluated by carrying out six independent determinations of FOR, TRI, and CLS (0.6 μg/mL of FOR, 0.9 μg/mL of TRI and 20 μg/mL of CLS) test samples against the qualified working standard. The method precision study shows the repeatability of the results obtained by the testing method. The % RSD (n=6) was 0.3 % for FOR, 0.5 % for TRI and 0.7 % for CLS, which are well within the acceptable limit of 2.0%. It was confirmed from results that the method is precise for the intended purpose (Table 7).

#### Intermediate precision: (Reproducibility)

The purpose of this study was to demonstrate the reliability of the test results with variations. The reproducibility was checked by analyzing the samples with a different analyst, using a different chromatographic system and column, on a different day. The analysis was conducted in the same manner as the method precision and the % RSD of all six sets of sample preparations was determined (Table 7). The % RSD for all three compounds is well within the acceptance criteria of 2.0%, so this study proved that the method is rugged enough for day to day use.

### Accuracy

The accuracy of an analytical method is the closeness of test results obtained by that method compared with the true values. To confirm the accuracy of the proposed method, recovery experiments were carried out by the standard addition technique. The accuracy of the method was carried out by adding known amounts of each drug corresponding to three concentration levels; 50, 100, and 150% of the label claim (Table 1) along with the excipients in triplicate. The samples were given the same treatment as described in the sample preparation. The percentage recoveries of FOR, TRI, and CLS at each level and each replicate were determined. The mean percentage recovery (n=3) and the relative standard deviation was calculated. The amount recovered was within ± 2.0% of the amount added, which indicates that there is no interference due to excipients present in the pressurized metered-dose inhaler. It was confirmed from results that the method is highly accurate (Table 8).

### Linearity

The linearity of an analytical method is its ability to elicit test results that are directly, or by a well-defined mathematical transformation, proportional to the concentration of analyte in a sample within a given range. The nominal concentrations of the standard and test solutions for FOR, TRI, and CLS were 0.6, 0.9 and 20 μg/mL, respectively. The response function was determined by preparing standard solutions at five different concentration levels ranging from 0.3–0.9 μg/mL for FOR, 0.45–1.35 μg/mL for TRI, and 10–30 μg/mL for CLS (50 to 150% of analyte concentration). The response was found to be linear from 50% to 150% of the standard concentration. The regression statistics are shown in Table 9.

### Robustness

The robustness of an analytical procedure is a measure of its capacity to remain unaffected by small, but deliberate variations in method parameters and provides an indication of its reliability during normal usage. The effect of change in flow rate (± 0.2 mL/min) and column oven temperature (± 5°C) on resolution (between FOR and TRI), theoretical plates, and tailing factor were studied. It was confirmed from results that the method is robust with respect to variability in above conditions (Table 10).

### Stability of sample in diluent

Drug stability in pharmaceutical formulations is a function of the storage conditions and chemical properties of the drug and its impurities. The condition used in stability experiments should reflect situations likely to be encountered during actual sample handling and analysis. Stability data is required to show that the concentration and purity of analyte in the sample at the time of analysis corresponds to the concentration and purity of analyte at the time of sampling. Stability of the sample solution was established by storage of the sample solution at ambient temperature (25°C) for 24 hours. The sample solution was re-analyzed after 12- and 24-hour time intervals, and assays were determined for the compounds (FOR, TRI, and CLS) and compared against the fresh sample. The sample solution did not show any appreciable changes in assay value when stored at ambient temperature up to 24 hours, which is presented in Table 11. The results from the solution stability experiments confirmed that the sample solution was stable for up to 24 hours during assay determination.

### Application of the method to dosage forms

The present method is applied to the estimation of drugs in the commercially available metered-dose inhaler (Table 12).

## Experimental

### Materials and Reagents

The working standards and placebo of the drug product were provided by Dr. Reddy’s laboratories Ltd., Hyderabad, India. HPLC grade acetonitrile was obtained from J. T. Baker (NJ, USA). GR grade perchloric acid was obtained from Qualigens Ltd. 0.45 μm nylon membrane filter, and nylon syringe filters were purchased from Pall Life Science limited (India). High purity water was generated by using Milli-Q Plus water purification system (Millipore^®^, Milford, MA, USA).

### Equipments

The Cintex digital water bath was used for the specificity study. The photostability study was carried out in a photostability chamber (SUNTEST XLS+, ATLAS, Germany). The thermal stability study was performed in a dry air oven (Cintex, Mumbai, India).

### Chromatographic conditions

Analyses were performed on the Waters 2487 HPLC^TM^ system (Waters, Milford, USA), consisting of a binary solvent manager, sample manager, and PDA (photo diode array) detector. System control, data collection, and data processing were accomplished using Waters Empower^TM^-2 chromatography data software. The chromatographic condition was optimized using the Zorbax SB C8, (150 × 4.6 mm, 5.0 μm) column. 0.2% v/v perchloric acid was used as solvent-A and acetonitrile was used as solvent-B. The separation of FOR, TRI, CLS, and all unknown impurities was achieved by gradient elution using solvent-A and B (Table 3). Mixture of solvent-A and solvent-B in the ratio of 50:50 (v/v), respectively, was used as a diluent. The final selected and optimized conditions were as follows: injection volume 100 μL, gradient elution (Table 3) at a flow rate of 1.2 mL/min, temperature at 25°C (column oven), and detection wavelength at 230 nm. Under these conditions, the backpressure in the system was about 1200 psi. The stress degraded samples and the solution stability samples were analyzed using a PDA detector covering the range of 200–400nm.

### Standard solution preparation

The standard solution was prepared by dissolving standard substances in diluent to obtain solution containing 0.6 μg/mL of FOR, 0.9 μg/mL of TRI, and 20 μg/mL of CLS.

### Sample solution preparation

The pressurized canister was removed from the actuator, and the label and ink were removed from the canister with a suitable solvent. The canister was wiped with tissue paper and then primed as per the priming procedure. The pressurized canister was removed from the actuator, and the stem and valve were cleaned by the diluent. Solvent was removed from the stem and valve using a narrow jet filter line. A stainless steel base plate (with three lags and a central circular indentation with a hole about 1.5 mm in diameter) kept inside the 100 mL glass beaker, had about 50 mL of diluent added to it, which insured that the discharge volume did not drop below 25mm under the surface of the solvent. The pressurized canister was shaken for 15 seconds in an inverted position. The canister was placed invertedly in the hole of the stainless steel base plate, discharged one spray, and then waited for two seconds. The container was shaken for at least five seconds. Once again, the canister was discharged. The same procedure was repeated another eight times. The pressurized container was removed and washed along with the valve and stem with five to ten mL of diluent. The washing solution was collected in the same 100 mL beaker. The entire beaker (holding solution from two to three washings) content was transferred to the 100 mL volumetric flask. This solution was then diluted up to the mark with diluent and mixed well.

### Placebo solution preparation

The placebo pressurized canister was taken and same procedure was followed as per sample preparation.

### Market product sample solution preparation (for Triohale Inhaler)

The sample solution preparation was followed for the marketed sample preparation.

## Conclusion

A rapid, RP-HPLC method was successfully developed for the simultaneous estimation of FOR, TRI, and CLS in a pressurized metered-dose inhaler. The developed method is selective, precise, accurate, linear, and robust. Forced degradation data proved that the method is specific for the analytes and free from the interference of placebo and unknown degradation products. The run time (10 min) enables rapid determination of the pressurized metered-dose inhaler. Moreover, it may be applied for individual and simultaneous determination of FOR, TRI, and CLS compound in the pharmaceutical drug product (pulmonary drug formulation) and drug substance. Also, it can be utilized for determination of assay, content uniformity (μg/spray), emitted dose by TSI (Twin stage impinger), and Aerodynamic particle size distribution by ACI (Anderson cascade impactor) of pulmonary drug products.

## Figures and Tables

**Tab. 1:** Formulation label claim with its working concentration

Compound	Formulation label claim per Spray	Working concentration in μg/mL
FOR	Formoterol Fumarate 6 mcg	0.6
TRI	Tiotropium bromide 9 mcg	0.9
CLS	Ciclesonide 200 mcg	20

**Tab. 2:** Summary of solvent used to optimize the method

Solvent-A	Solvent-B	Observation
Water	Acetonitrile	Broad peaks were observed
0.05% v/v H_3_PO_4_	Acetonitrile	Non symmetric peaks and unsatisfactory resolution (between FOR and TRI)
Water (pH 3.5 with HClO_4_)	Acetonitrile	Non Symmetric peaks and unsatisfactory resolution (between FOR and TRI)
0.2% v/v HClO_4_	Acetonitrile	Symmetric peaks and satisfactory resolution (between FOR and TRI)

**Tab. 3:** Gradients program for elution

Time (min)	Flow rate (mL/min)	% Solvent-A	% Solvent-B	Curve
Initial	1.2	70	30	Isocratic
0.1	1.2	70	30	Isocratic
3.0	1.2	0	100	Linear
9.0	1.2	0	100	Linear
9.1	1.2	0	100	Isocratic
10.0	1.2	70	30	Equilibration

**Tab. 4:** Summary of stationary phase used to optimize the method

Stationary phase	Dimension	Observation/Remarks
Zorbax SB C18	(150 × 4.6) mm, 5 μm	Peak merging (FOR and TRI)
Inertsil ODS-3V	(150 × 4.6) mm, 5 μm	Poor resolution (FOR and TRI)
Zorbax SB C8	(150 × 4.6) mm, 5 μm	Symmetric peaks and satisfactory resolution (FOR and TRI)

**Tab. 5:** Summary of forced degradation results

Stress condition	Observation
Control sample	Not applicable
Acid hydrolysis (0.1N HCl, 60°C, 1h)	Formation of unknown impurity (at RT 1.397)
Alkaline hydrolysis (0.01N NaOH, Rt, 1h)	Formation of two unknown impurities (at RT 1.411 and 2.589)
Oxidation (30% H_2_O_2_, rt, 1h)	Formation of unknown impurity (at RT 6.344)
Thermal (60°C, 1h)	Unknown impurity not observed
Photolytic (1.2 million Lux hours)	Formation of unknown impurity (at RT 3.731)

rt… Room temperature; RT… Retention time

**Tab. 6:** System suitability results (precision and intermediate precision)

Test	Parameters	FOR	TRI	CLS	Proposed criteria
Precision	USP resolution	–	1.7	–	NLT 1.5
	USP tailing	1.0	1.1	1.1	NMT 1.5
	USP plate count	2687	4678	9731	NLT 2000
	Area % RSD*	0.3	0.2	0.1	NMT 2.0%
Intermediate precision	USP resolution	–	1.7	–	NLT 1.5
	USP tailing	1.1	1.0	1.1	NMT 1.5
	USP plate count	2700	4690	9641	NLT 2000
	Area % RSD*	0.4	0.3	0.2	NMT 2.0%

USP… United States Pharmacopoeia; NLT… Not less than; NMT… Not more than;

* … Determined on six values

**Tab. 7:** Precision and Intermediate precision results

Substance	Precision at 100%		Intermediate precision	

	% Assay #	% RSD*	% Assay #	% RSD*
FOR	101.1	0.3	101.0	0.4
TRI	99.3	0.5	99.5	0.6
CLS	98.1	0.7	98.3	0.9

# ... Average of six determinations;

* … Determined on six values.

**Tab. 8:** Accuracy results

Substance	At 50% (n=3)		At 100% (n=3)		At 150% (n=3)	

	%Recovery	%RSD	%Recovery	%RSD	%Recovery	%RSD

FOR	100.1	0.5	99.3	0.4	99.0	0.4
TRI	99.2	0.4	99.6	0.5	100.5	0.4
CLS	100.2	0.4	99.3	0.7	99.7	0.5

**Tab. 9:** Regression statistics

Compound	Linearity range (μg/mL)	Correlation coefficient (r^2^)	Linearity (Equation)	Y-intercept bias in %
FOR	0.3 to 0.9	0.999	y = 22194x + 4894.7	0.220
TRI	0.45 to 1.35	0.999	y = 1133x + 2191.9	1.894
CLS	10.0 to 30.0	0.999	y = 1125.5x + 770.91	0.681

**Tab. 10:** Robustness study results

Condition	Parameters	FOR	TRI	CLS	Proposed criteria
Precision	USP resolution	–	1.7	–	NLT 1.5
	USP tailing	1.0	1.1	1.1	NMT 1.5
	USP plate count	2687	4678	9731	NLT 2000

At flow rate 1.0 mL/min	USP resolution	–	1.8	–	NLT 1.5
	USP tailing	1.1	1.2	1.1	NMT 1.5
	USP plate count	2350	4289	9503	NLT 2000

At flow rate 1.4 mL/min	USP resolution	–	1.6	–	NLT 1.5
	USP tailing	1.0	1.1	1.0	NMT 1.5
	USP plate count	2713	4820	9929	NLT 2000

At 20°C column oven temperature	USP resolution	–	1.7	–	NLT 1.5
	USP tailing	1.1	1.1	1.1	NMT 1.5
	USP plate count	2730	4701	9894	NLT 2000

At 30°C column oven temperature	USP resolution	–	1.6	–	NLT 1.5
	USP tailing	1.0	1.1	1.1	NMT 1.5
	USP plate count	2798	4725	10050	NLT 2000

**Tab. 11:** Solution stability results

Time intervals	FOR	TRI	CLS
% Assay Initial	100.5	99.5	98.0
% Assay after 12h	100.2	99.8	98.3
% Assay after 24h	99.9	99.3	98.5

**Tab. 12:** Result of market product (Triohale^®^, Cipla Ltd.)

Product Name and Labeled claim (in μg/spray)	FOR μg/spray	TRI μg/spray	CLS μg/spray
Triohale® [FOR(6 μg); TIO(9 μg); CLS (200 μg) ]	6.02	9.02	203.1
